# Strategies to Maintain Natural Biocontrol of Soil-Borne Crop Diseases During Severe Drought and Rainfall Events

**DOI:** 10.3389/fmicb.2018.02279

**Published:** 2018-11-02

**Authors:** Annelein Meisner, Wietse de Boer

**Affiliations:** ^1^Microbial Ecology Group, Department of Biology, Lund University, Lund, Sweden; ^2^Department of Microbial Ecology, Netherlands Institute of Ecology (NIOO-KNAW), Wageningen, Netherlands; ^3^Department of Soil Quality, Wageningen University and Research Centre, Wageningen, Netherlands

**Keywords:** extreme weather events, climate change, crop, pathogen, disease suppression, soil microorganisms, antagonistic interactions

## Abstract

In many parts of the world, agricultural ecosystems are increasingly exposed to severe drought, and rainfall events due to climate changes. This coincides with a higher vulnerability of crops to soil-borne diseases, which is mostly ascribed to decreased resistance to pathogen attacks. However, loss of the natural capacity of soil microbes to suppress soil-borne plant pathogens may also contribute to increased disease outbreaks. In this perspectives paper, we will discuss the effect of extreme weather events on pathogen-antagonist interactions during drought and rainfall events and upon recovery. We will focus on diseases caused by root-infecting fungi and oomycetes. In addition, we will explore factors that affect restoration of the balance between pathogens and other soil microbes. Finally, we will indicate potential future avenues to improve the resistance and/or recovery of natural biocontrol during, and after water stresses. As such, our perspective paper will highlight a knowledge gap that needs to be bridged to adapt agricultural ecosystems to changing climate scenarios.

## Introduction

Climate change is expected to increase the exposure of agricultural ecosystems to extreme drought and rainfall events (IPCC, 2012; Fischer and Knutti, 2016), which can result in severe decreases in crop yields (Challinor et al., 2014; Obidiegwu et al., 2015; Challinor et al., 2016; Eurostats, 2016). It will, therefore, be a great challenge to maintain sufficient food production for the growing human population. Next to direct decreases in crop yields due to unfavorable growth conditions, additional problems may be caused by a reduced resistance of agricultural crops to soil-borne plant pathogen attacks after drought and rainfall events (Ramegowda and Senthil-Kumar, 2015; Dikilitas et al., 2016). The coincidence of extreme weather events and higher vulnerability of crops to pathogen attacks can be due to a decrease in the plant immune response (for a detailed review on this topic see Ramegowda and Senthil-Kumar, 2015) and/or an altered pathogen pressure.

Root-infecting fungi and oomycetes are two major groups of pathogens causing problems in agricultural crops at a broad range of moisture levels (Duncan and Kennedy, 1989; Dixon and Tilston, 2010; Thompson et al., 2013). For example, high water content increases the ability of motile zoospores of plant pathogenic oomycetes to reach roots (Malajczuk and Theodorou, 1979; Judelson and Blanco, 2005). In contrast, drought increases the amount of drought resistant microorganisms. Fungi are often more resistant to drought than bacteria (Barnard et al., 2013; Meisner et al., 2013; de Vries et al., 2018) and many fungal pathogens, such as species belonging to *Fusarium* or *Verticillium* genera, are often involved in increased pathogen pressure during drought, (Dikilitas et al., 2016). Hence, the types of pathogens that thrive under drought and wet conditions will differ.

A largely ignored potential mechanism of increased pathogen pressure after an extreme drought or rainfall event is the reduction of the natural capacity of soil to suppress pathogens. The legacy of an environmental stress, including water stress, can decrease the biological suppression of crop pathogens and therewith increase the vulnerability of crops for pathogen attacks (Ho and Ko, 1985; Lootsma and Scholte, 1997; van Agtmaal et al., 2015). Most soils show a certain level of suppression against pathogenic fungi and oomycetes, often referred to as general soil suppression (Garbeva et al., 2011). Competitive interactions in soil microbial communities are thought to be the major causal factor of general soil suppression (Garbeva et al., 2011). In addition, some soils show so-called specific suppression against one pathogenic species (Raaijmakers and Mazzola, 2016). The plant’s response to increased pathogen abundance depends on the microbial community colonizing the roots and the plant’s ability to tolerate water stress. The colonization of plant roots by soil microorganisms is influenced by the amount and composition of rhizodeposits (Philippot et al., 2013). Several root-colonizing microorganisms are known to improve the plants response to pathogens (Berendsen et al., 2012). In addition, several rhizosphere microorganisms can increase drought tolerance in plants (Ngumbi and Kloepper, 2016). However, there is limited information about interactions of plant-growth promoting microbes with pathogens during drought stress and upon recovery. In this perspectives paper, we propose that improvements to the maintenance and recovery of suppression of plant pathogens during and after drought and rainfall may prevent severe losses due to soil-borne pathogens. In addition, we will suggest areas for future research that improve our understanding of how extreme drought and rainfall events will affect interactions between pathogen suppressive microorganisms and crop pathogens.

## Antagonistic Interactions Between Pathogens and Heterotrophic Microbes

**FIGURE 1:**
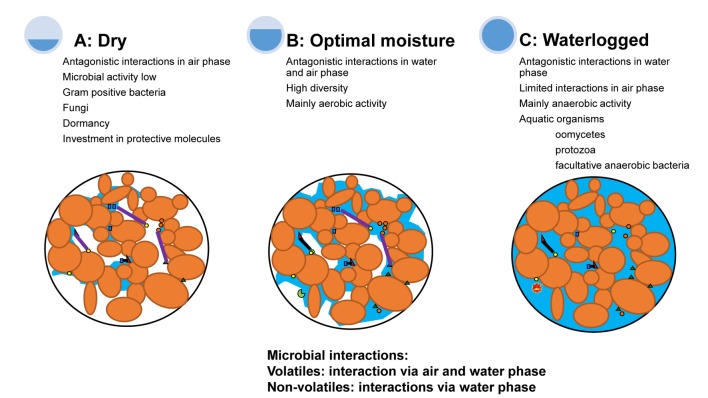
The types of antagonistic interactions between pathogens and other soil microorganisms are influenced by water availability. Under dry conditions **(A)**, there is a big air phase and the interactions between microorganisms may occur mainly via volatile organic compounds in the air phase. However, the microbial activity of both resident and plant pathogens is low when moisture is limiting. Microorganisms that survive drought may invest in protective molecules or formation of dormancy structures. During optimal moisture conditions **(B)**, most microorganisms grow aerobically and interact via secondary chemicals, enzymes and volatiles in both the air and water phase. During waterlogged conditions **(C)**, interactions between microorganisms occur in the water phase of soils. Microorganisms that survive waterlogged conditions include organisms that can cope with anoxic conditions. Small triangles, squares, and circles reflect different soil microorganisms. The purple lines reflect microbial interactions that occur in the air phase and the black lines reflect interactions that occur in the water phase of soil. The blue areas indicate the water phase and the white areas the air phase. Figure adapted from Moyano et al. (2013).

The suppression of pathogen infection on roots is caused by interactions with other soil microorganisms (van Os et al., 1999; Duran et al., 2017) and often occurs via the production of inhibitory secondary metabolites (Garbeva et al., 2011). Chemical compounds, such as antibiotics, that are produced during antagonistic interactions between competing heterotrophic microbes may also affect other biota in soils, including pathogens (Garbeva et al., 2011; Raaijmakers and Mazzola, 2012; Schulz-Bohm et al., 2017). Most secondary chemicals exuded by microorganisms can diffuse through the water-filled area of soil pores and, therefore, only interact with microbes that live in the water phase (Tyc et al., 2017). However, one group of secondary compounds, volatiles, is of special interest, as volatiles can diffuse through both the water-filled and air-filled soil pores thereby widening the spatial range of inhibition of pathogens (Schmidt et al., 2015; Tyc et al., 2017). As such, the impact of fluctuations of soil water content on the role of volatiles in pathogen suppression is of special interest (Peñuelas et al., 2014). Differences in moisture content will affect the composition of chemical compounds produced by soil microbes (Bastos and Magan, 2007; Hiltpold and Turlings, 2008). Waterlogged conditions after heavy rainfall will expel gasses from soil and reduce the movement of gasses in soil (Moyano et al., 2013). Volatiles will be especially involved in competitive interactions in the air-filled area of the pores in unsaturated soils (Figure 1A), whereas water soluble secondary metabolites will be the main compounds in antagonistic interactions during waterlogged conditions (Figure 1C). Therefore, the chemical and physical characteristics of secondary metabolites that are effective in suppressing interactions will be determined by soil moisture conditions (Figure 1).

There is increasing evidence that volatiles produced by soil microorganisms play an important role in the natural suppression of pathogens. For example, growth of three common plant pathogens was inhibited by volatiles emitted from 50 agricultural soils (van Agtmaal et al., 2018). Production of pathogen-inhibiting volatiles by bacteria has received particular attention in research (Garbeva et al., 2011; Schmidt et al., 2015; Schulz-Bohm et al., 2017). Research on suppression of fungal pathogens by bacterial volatiles has also indicated that composition of bacterial communities is an important determinant of the spectrum of volatiles produced. For example, loss of rare soil bacteria decreased volatiles that suppressed *in vitro* growth of the plant pathogen *Fusarium oxysporum* (Hol et al., 2015). In addition, the legacy of anaerobic disinfection, which is the anaerobic treatment of soil in between crop cover, reduced volatiles and pathogen suppression three months after recovery, via effects on the bacterial community composition (van Agtmaal et al., 2015). Differences in soil moisture can affect the composition of the microbial community (Barnard et al., 2015; Hartmann et al., 2017; Meisner et al., 2018) and, consequently, also the spectrum of inhibiting compounds. The question remains if these changes coincide with altered pathogen suppression.

## Balance Between Soil Pathogens and Heterotrophic Soil Microbes

**FIGURE 2:**
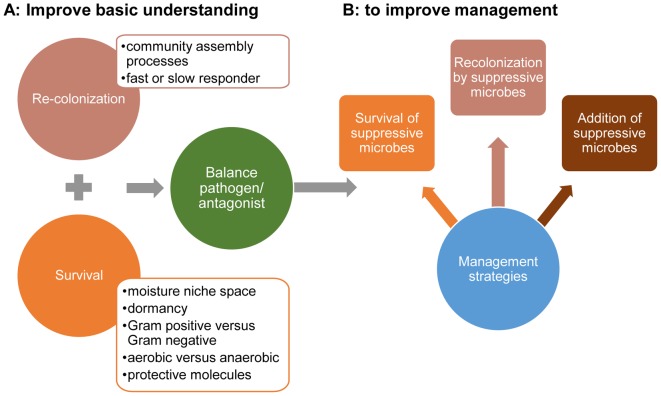
Future research priorities are to improve basic understanding of microbial interactions that affect the balance between pathogens and antagonists upon their survival during exposure to extreme water stress and recolonization strategies during moisture stress and upon recovery **(A)** and use this basic understanding to improve management strategies that improve the pathogen suppression **(B)**.

Pathogen suppression will be influenced by the response of both heterotrophic microorganisms and pathogens to drought and waterlogged conditions as well as their ability to recover (Figure 2A). First, both pathogens and heterotrophic microorganisms have to survive the extreme conditions. This will likely depend upon the niche space for water availability as microbial species, including pathogens, differ in their potential to maintain activity along a range of matric potentials (Whiting et al., 2001; Lennon et al., 2012). A wider niche space for a microorganism results in a higher chance of surviving the extreme conditions and, consequently, a higher chance to be present in the recovery phase. Soil microorganisms often experience anoxic conditions when exposed to waterlogged conditions. This can have an impact on the composition of microbes in the recovery phase (van Agtmaal et al., 2015). Microbes may also survive unfavorable conditions by going into dormancy (Manzoni et al., 2014; Shoemaker and Lennon, 2018), by producing protective molecules, such as osmolytes (Warren, 2014) or extracellular peptides (Or et al., 2007). Another strategy to survive is to have a thicker cell wall such as the thick peptidoglycan layer of Gram positive bacteria (Potts, 1994; Schimel et al., 2007).

Although there are many survival strategies to cope with drought and waterlogged conditions, cells of many soil microorganisms are irreversibly damaged (Nocker et al., 2012). For example, drying increases damage to DNA and enzymes (Dose et al., 1991; Potts, 1994). As a result, the active microbial biomass size is reduced upon recovery (Kieft et al., 1987; Lennon et al., 2012; Meisner et al., 2017). The partial elimination of microbes does result in an increase in the number of empty niches available upon recovery that both pathogens and other microbes can colonize. The success of colonization of empty niches by microbial species is determined by community assembly rules, such as priority effects. Priority effects describe the inhibitory or facilitative effects of early arriving species on next arriving ones (Fukami, 2015). Species that will recover faster from an extreme weather event will likely have a priority to become abundant first (Placella et al., 2012). In addition, dispersal due to movement of spores via wind or mixing of the content of soil pores during heavy rainfall and rewetting events can affect the composition of microbial species developing during recovery (Szekely and Langenheder, 2017).

The increased availability of easily available substrates upon recovery (Williams and Xia, 2009) due to increased necromass will act as a surplus of food sources for both pathogens and other microorganisms. This implies that the competitive pressure for energy resources is temporarily relieved. This is expected to coincide with a decrease in intensity of antagonistic interactions between microorganisms, including antagonistic interactions that suppress soil-borne plant pathogens. A similar condition can be created by adding easily available substrates to soils. For example non-mature compost can result in an increased infection by soil-borne pathogens (Hoitink and Grebus, 1994). Several factors can contribute to an increased risk for outbreaks of soil-borne pathogens during nutrient excess, namely (1) lower colonization of microorganisms that suppress pathogens (Hoitink et al., 1997); (2) decreased production of secondary metabolites due to investment of nutrients in growth and not in defense strategies (Coley et al., 1985; de Boer et al., 2003; Ghoul and Mitri, 2016); (3) reduced sensitivity of microorganisms, which are well fed, to inhibitory compounds, because they invest more in defense strategies (Garbeva et al., 2011). Thus, community assembly processes, the availability of labile nutrients and empty niches will influence the composition of the microbial communities during the recovery phase. Indeed, composition of microbial communities has often been observed to differ with different moisture treatments (Fierer et al., 2003; Drigo et al., 2017; Hartmann et al., 2017; Naylor and Coleman-Derr, 2017). In summary, water-related stress due to drought and rainfall events will change the interactions between microorganisms, which will affect the opportunities of pathogens to infect roots.

## Future Research to Improve Agricultural Adaptation to Climate Change

Future research should take into account knowledge about microbial interactions, survival, and recovery of pathogens and antagonistic microorganisms during or after extreme water stress events to find strategies for increasing pathogen suppressive activities of microbes (Hawkes and Connor, 2017). Most important is to have insight in the key factors that affect the balance between heterotrophic soil microbes and pathogens. In this section, we will indicate knowledge gaps and management strategies that could be explored for the improvement of pathogen suppression upon the recovery of agricultural soil after drought or rainfall events.

### Knowledge Needed to Improve Survival of Pathogen Suppressive Microorganisms

Survival of microorganisms is dependent on the moisture niche space and microbial traits (See “Balance Between Soil Pathogens and Heterotrophic Soil Microbes”). There are indications that drought is a natural selector for the microbial community, as microbial communities differ in soil with a legacy of drought, weeks to months after recovery (Bouskill et al., 2013; Meisner et al., 2018). Changes in the microbial community composition after a stress can affect the response of the microbial community to an additional drought stress. For example, microbial communities with a drought legacy seem to have a better ability to cope with an additional drought than microorganisms previously exposed to ambient conditions (Evans and Wallenstein, 2014). In addition, drought adapted microbes can improve fitness of plant species exposed to dry conditions (Lau and Lennon, 2012; Ngumbi and Kloepper, 2016). Drought-adapted microbes do not only improve the drought tolerance of their host plant, but also of other plants (Rodriguez et al., 2008; Marulanda et al., 2009). Drought exposed microorganisms can also recover faster to other stresses (van Kruistum et al., 2018). However, the question remains if drought-tolerant microorganisms suppress pathogens.

Microorganisms that survive waterlogged conditions need to cope with a wide range of oxygen concentrations (Neira et al., 2015). For example, *Enterobacteriaceae* have been observed to maintain metabolic activity when going from oxic to anoxic conditions after a rainfall event (Degelmann et al., 2009). In addition, a legacy of waterlogged conditions, such as flooding can result in a reduced suppression of bulb-rot causing *Pythium* spp. (van Os et al., 1999). The anaerobic activity of microbes is releasing compounds like organic acids, organic sulfides, and ammonia that can be toxic to aerobic microbes. This is the reason why stimulation of anaerobic decomposition of incorporated organic material into agricultural soils is used as a method to kill aerobic pathogens (Strauss and Kluepfel, 2015). However, changes in microbial community composition due to anaerobic disinfestation can cause a drastic reduction of the pathogen suppressive capacity of soils that remains present months after recovery (van Agtmaal et al., 2015). This implies that pathogens that will survive waterlogged conditions can remain abundant in the recovery phase. However, it is unknown if microorganisms that survive anaerobic conditions can improve pathogen suppression upon a second rainfall event.

### Strategies to Improve Re-colonization of Pathogen Suppressive Microbes

Management strategies should focus on ways to improve re-colonization of empty niches by microbes that suppress pathogens, as this would allow for an earlier recovery of pathogen suppression. One way of improving recovery is the addition or manipulation of organic material, as the ‘carrying capacity of substrate’ has been suggested to regulate species composition, their abundance, and activity and therewith regulates the suppression of pathogens (Hoitink et al., 1997). Soil with higher carbon content can maintain higher moisture levels during droughts (Ng et al., 2015) and higher microbial biomass (Hueso et al., 2012). Accordingly, the addition of organic material may improve survival and create patches of microbes that can colonize empty niches upon recovery. However, difference in decomposition stage of the organic material can be important to consider. Early stages of the breakdown of organic material have many easily available substrates and are low in supporting pathogen suppression. In contrast, later stages with more recalcitrant substrates may have higher pathogen suppression (Hoitink et al., 1997; Bonanomi et al., 2010; Berg and McClaugherty, 2014). Differences in decomposition stage may explain why organic amendments can have different effects on the microbial biomass after recovery (Bapiri et al., 2010; Lado-Monserrat et al., 2014; Ng et al., 2015). As such, there are many avenues for future studies to identify if and how patches of organic material affect pathogen suppression during the recovery phase.

Pathogen suppression could also be managed by the addition of specific microorganisms or complete microbial communities (O’Hanlon et al., 2012). For example, the addition of a forest fungus (*Penicillium* WPTIIIA3) can increase yields of winter wheat when this species is exposed to drought and *Fusarium* pathogens (Ridout and Newcombe, 2016). This strategy would be beneficial when knowledge of the specific pathogen and pathogen suppressive microorganism is available (Borneman and Becker, 2007). However, added single strains need to establish and overcome the colonization resistance of the soil microbiome (van Veen et al., 1997; de Boer, 2017), which can be difficult due to the high diversity of soil microbial communities (van Elsas et al., 2012; Bashan et al., 2014). Thus, it can be difficult to overcome the colonization resistance of the resident community when all niches are filled with other microbes. These difficulties can change when extreme weather events result in empty niches for the introduced microorganism to establish. Therefore, the addition of beneficial microorganisms in the recovery phase may be successful as they can colonize empty niches and can be worthwhile to be investigated (Adam et al., 2016). The addition of beneficial microbes could potentially be combined by rewetting with water spraying systems during the recovery from drought conditions. An alternate strategy could be to engineer microbial communities that benefit host plants under climate change, suppress pathogens and are able to colonize, and survive in the soil environment (Oyserman et al., 2018). These beneficial microorganisms could belong to the group of plant growth promoting microorganisms as they have the ability to both improve the plants physiological response to drought in sterile soils (Mayak et al., 2004; Timmusk et al., 2014) and can act as disease control agent (Kloepper et al., 2004). However, future studies should identify plant growth promoting microorganisms that can both improve drought resistant and disease resistance in crops (Coleman-Derr and Tringe, 2014; Ngumbi and Kloepper, 2016).

## Conclusion

We conclude that the higher sensitivity of crops to infections by soil-borne pathogens during and after extreme weather events is in part due to loss of the pathogen suppressive capacity of soils. Therefore, adaptation of agricultural ecosystems to changing climate scenarios should include improvements of pathogen suppression of soil during and after extreme drought and rainfall events. However, basic knowledge about effects of extreme weather events on microbial interactions, survival of microorganisms that induce pathogen suppression as well as recovery of the pathogen suppression appears not to be addressed in literature. This knowledge is needed to develop management strategies that improve pathogen suppressive soils (Figure 2). Management strategies should focus on improving survival and early recolonization of pathogen-suppressing microorganisms during the recovery phase after extreme weather events. Improved survival may be achieved via the natural selection of soil microorganisms to cope with drought or waterlogged conditions (selection by repeated stress) or via the addition of organic materials (survival spots). The challenge will be to find a strategy that allows to manage both drought and waterlogged conditions as the microorganism that respond to drought will differ from the ones that survive waterlogged conditions. In addition, improved and faster recovery of pathogen suppressive microorganisms can be managed by the addition of pathogen suppressive microorganisms. As such, there are many research directions to improve our understanding of pathogen suppression during and upon recovery to the drought and rainfall events. This understanding is needed to adapt agricultural ecosystems to changing climate scenarios.

## Author Contributions

AM and WB conceived and designed the ideas for the article and wrote the manuscript.

## Conflict of Interest Statement

The authors declare that the research was conducted in the absence of any commercial or financial relationships that could be construed as a potential conflict of interest.
